# NADPH Oxidase Hyperactivity Contributes to Cardiac Dysfunction and Apoptosis in Rats with Severe Experimental Pancreatitis through ROS-Mediated MAPK Signaling Pathway

**DOI:** 10.1155/2019/4578175

**Published:** 2019-05-09

**Authors:** Yi Wen, Ruohong Liu, Ning Lin, Hao Luo, Jiajia Tang, Qilin Huang, Hongyu Sun, Lijun Tang

**Affiliations:** ^1^Department of Graduate School, The Third Military Medical University (Army Medical University), Chongqing, China; ^2^Department of General Surgery & Pancreatic Injury and Repair Key Laboratory of Sichuan Province, The General Hospital of Western Theater Command (Chengdu Military General Hospital), Chengdu, China; ^3^Department of Ultrasound, Chinese Academy of Medical Sciences and Peking Union Medical College Hospital, Beijing, China

## Abstract

NADPH oxidase (Nox) is considered a major source of reactive oxygen species (ROS) in the heart in normal and pathological conditions. However, the role of Nox in severe acute pancreatitis- (SAP-) associated cardiac injury remains unclear. Therefore, we aim to investigate the contribution of Nox to SAP-associated cardiac injury and to explore the underlying molecular mechanisms. Apocynin, a Nox inhibitor, was given at 20 mg/kg for 30 min before SAP induction by a retrograde pancreatic duct injection of 5% sodium taurocholate. Histopathological staining, Nox activity and protein expression, oxidative stress markers, apoptosis and associated proteins, cardiac-related enzyme indexes, and cardiac function were assessed in the myocardium in SAP rats. The redox-sensitive MAPK signaling molecules were also examined by western blotting. SAP rats exhibited significant cardiac impairment along with increased Nox activity and protein expression, ROS production, cell apoptosis, and proapoptotic Bax and cleaved caspase-3 protein levels. Notably, Nox inhibition with apocynin prevented SAP-associated cardiac injury evidenced by a decreased histopathologic score, cardiac-related enzymes, and cardiac function through the reduction of ROS production and cell apoptosis. This protective role was further confirmed by a simulation experiment *in vitro.* Moreover, we found that SAP-induced activation in MAPK signaling molecules in cardiomyocytes was significantly attenuated by Nox inhibition. Our data provide the first evidence that Nox hyperactivation acts as the main source of ROS production in the myocardium, increases oxidative stress, and promotes cell apoptosis via activating the MAPK pathway, which ultimately results in cardiac injury in SAP.

## 1. Introduction

Severe acute pancreatitis (SAP) is a fatal systemic disease characterized by rapid progression and high mortality, and it is frequently complicated with injury of distant organs, including the lungs, intestine, kidneys, and heart [1, 2]. Among them, SAP-associated cardiac injury occurs alone or simultaneously with other organ injuries in all stages of SAP [3]. To data, several mechanisms are reportedly involved in SAP-associated cardiac injury including metabolic changes, circulating proteolytic enzymes, and systemic inflammatory response [4]. Despite advances in our understanding of the pathophysiology of SAP-associated cardiac injury, the exact mechanisms underlying the disease have yet to be fully elucidated.

Numerous studies have revealed that the increase in reactive oxygen species (ROS) production contributes to the development of cardiac diseases such as cardiac hypertrophy, myocardial infarction, and heart failure [5, 6]. The nicotinamide adenine dinucleotide phosphate (NADPH) oxidase (Nox) is considered the main source of reactive oxygen species (ROS) in the cardiovascular system [7]. The Nox family is a multicomponent enzyme, comprised of seven members including Nox1-5 and Duox 1 and 2. Of these, Nox2 and Nox4 are highly expressed in the cardiomyocytes mediating both adaptive and maladaptive changes in the heart [8]. Nox activity is reported to be elevated in cardiac dysfunction under different disease states including sepsis, ischemic cardiomyopathy, and heart failure [9, 10]. Increasing evidence has shown that Nox is activated by various stimuli like proinflammatory cytokine TNF-*α*, endothelin-1 (ET-1), and endotoxin [11–13]. Once activated, Nox will mediate ROS generation, impair endogenous antioxidant capacity, cause oxidative stress damage, and ultimately lead to cardiac dysfunction [14]. Thus, an increase in ROS production is regarded as a major pathogenic factor in triggering cardiac dysfunction. However, whether Nox is involved in SAP-associated cardiac injury remains to be investigated.

As is known, cardiomyocyte apoptosis is the major pathological indicator of cardiac injury [15, 16]. Nox-derived ROS have been demonstrated to induce cardiomyocyte apoptosis, which causes the loss of contractile tissue and initiates cardiac remodeling [17]. As such, inhibition of Nox2 and Nox4 blocks ROS production, suppresses cardiomyocyte apoptosis, and ameliorates cardiac dysfunction and remodeling [18, 19]. Studies have also shown that the redox-sensitive mitogen-activated protein kinase (MAPK) signaling pathways are involved in cardiomyocyte apoptosis triggered by Nox-derived ROS [20]. Petrich and Wang reported that activation of JNK and p38 plays a critical role in myocardial hypertrophy and apoptosis [21]. However, whether the ROS-mediated MAPK signaling pathways are involved in SAP-induced cardiomyocyte apoptosis is unknown.

Based on these findings, we hypothesized that Nox would be hyperactivated in the heart of SAP rat models, contributing to increased oxidative stress and cell apoptosis, ultimately resulting in SAP-associated acute cardiac injury.

## 2. Materials and Methods

### 2.1. Reagents

Apocynin was supplied by Selleck Chemicals (Houston, TX, USA). Sodium taurocholate was purchased from Sigma-Aldrich (St. Louis, MO, USA). Antibodies specific for NADPH oxidase 2 (Nox2/gp91phox, ab31092), NADPH oxidase 4 (Nox4, ab133303), Bax (ab32503), p38 (ab170099), p-p38 (Thr180/Tyr182, ab4822), ERK1/2 (ab184699), p-ERK1/2 (Thr202/Tyr204 and Thr185/Tyr187, ab76299), and p-JNK (Thr183/Thr221, ab124956) were purchased from Abcam (Cambridge, MA, USA). Antibody for caspase-3 (# 9662S) was purchased from Cell Signaling Technology (Beverly, MA, USA). Antibody for Bcl-2 (GTX100064) was purchased from GeneTex Inc. (Irvine, CA, USA). Antibodies for JNK and GAPDH were purchased from Beijing Biosynthesis Biotechnology Co. Ltd. (Beijing, China). NADPH-Na4 was supplied by Beijing Solarbio Science and Technology Co. Ltd. (Beijing, China). Dihydroethidium (DHE) was purchased from Beyotime Biotechnology (Shanghai, China). All other chemicals used in this study were of analytical grade and were commercially available.

### 2.2. Animals and Experimental Design

Adult male Sprague Dawley rats (200-220 g) were used in this study. The animals were purchased from DaShuo Animal Science and Technology Co. Ltd. (Chengdu, China). They were kept separately in a system of individually ventilated cages (IVC) and fed with standard laboratory food and water ad libitum for 3 days before the experiments. Animals were fasted overnight before the experiment but had free access to water. Experimental procedures were approved by the Institutional Animal Care and Use Committee at the General Hospital of Western Theater Command and carried out in accordance with the established International Guiding Principles for Animal Research.

Briefly, anesthetization of rats was performed with 5% isoflurane (via an induction box) prior to surgery. A total of 60 SD rats were randomly divided into four groups: the sham operation (SO) group, the SAP group, the SAP+apocynin (SAP-APO) group, and the apocynin control (APO-CON) group (*n* = 15 for each group). The SAP model was induced by a standardized pressure-controlled retrograde infusion of 5% sodium taurocholate into the biliopancreatic duct at a rate of 12 mL/h by using a microinfusion pump (0.13 mL/100 g rat weight) and maintained for 5 min after injection; then, the microvascular clamp and puncture needle were removed, and the abdomen was then closed according to the classical method of Chen et al. with a little modification [22]. In the SO and APO-CON groups, an incision was made in the abdomen of the rats and was subsequently closed. Following the operation, all rats received 4 mL/100 g body weight of sterile saline every 6 h by subcutaneous injection in the back to compensate for anticipated fluid loss. In the SAP-APO group, 10% dimethyl sulfoxide (DMSO) containing apocynin (20 mg/kg) was applied by tail vein injection 30 min before SAP induction. The dosage of apocynin was selected on the basis of previous studies, which confirmed the *in vivo* effectiveness and absence of side effects in experimental models [23, 24]. In the SO and SAP groups, 10% DMSO solution (2 mL/kg) was administered 30 min prior to the operation. In the APO-CON group, 10% DMSO containing apocynin (20 mg/kg) was applied 30 min prior to the sham operation.

In *in vitro* studies, SAP rat serum was obtained from the sodium taurocholate-injected rats that met the following criteria: (1) serum cardiac enzyme abnormalities more than two times the normal upper limits and (2) myocardial cell apoptosis confirmed by immunofluorescence histochemistry analysis. Normal rat serum was obtained from the rats in the SO group. Cells were divided into four groups: the NS group was cultured with 10% normal rat serum medium, the SS group was cultured with 10% SAP rat serum medium, the SA group was cultured with 10% SAP rat serum medium and apocynin, and the NA group was cultured with 10% normal rat serum medium and apocynin.

### 2.3. Echocardiography and Hemodynamic Analysis

After the 24 h induction of SAP, all rats were anesthetized by 5% isoflurane, and echocardiography analysis was assessed by using an ultrasound apparatus (LOGIQ E9; GE Healthcare, Boston, USA) equipped with a 12 MHz transducer. Left ventricular end-diastolic dimension (LVEDD) and left ventricular end-systolic dimension (LVESD) were measured, and left ventricular ejection fraction (LVEF) and fractional shortening (FS) were calculated from M-mode recordings. Measurements were analyzed by a blinded observer, and all the results were averaged from five consecutive cardiac cycles measuring from the M-mode images. In the process of hemodynamic measurements, a microtip catheter transducer (22G IV cannula, Chengdu Xinjin Shifeng Medical Apparatus & Instrument Co. Ltd., Chengdu, China) was gradually inserted into the right carotid artery for 2 cm. The signals were continuously recorded using the ADInstruments PowerLab system (cat. no. PL3516; ADInstruments Pty Ltd., Bella Vista, Australia). The heart rate, systolic pressure (mmHg), and diastolic pressure (mmHg) were processed using LabChart 7 (v7.3.7) analysis software. After completing all measurements under strict aseptic conditions, blood samples were collected from the aorta abdominalis, serum was obtained by centrifugation at 3000 rpm for 15 min at 4°C, and an appropriate number of aliquots were separated and stored at –80°C until assaying. Then, the pancreas and hearts were quickly removed, and parts taken from the pancreas and the left ventricle were fixed in 4% paraformaldehyde or flash-frozen in liquid nitrogen until experimental use.

### 2.4. Histological Assessment

24 h after SAP induction, the terminal pancreas and heart tissue samples were paraformaldehyde-fixed, paraffin-embedded, and sectioned at 4 *μ*m. The sections were then stained with hematoxylin and eosin. The slides were read by a consultant histopathologist blinded to the groups using a Leica DM3000 light microscope (Leica Microsystems CMS GmbH, Wetzlar, Germany) with a digital photographic system (Leica application suite, version 4.4.0). For the heart, the histological scoring was evaluated based on a 0-3 scoring method as described previously [25] and the parameters included interstitial edema, hemorrhage, and neutrophil infiltration. The severity of pancreatic injury was evaluated based on a 0-4 scoring method and the parameters included edema, fat necrosis, hemorrhage, inflammatory cell infiltrate, and acinar necrosis [26].

### 2.5. Biochemical Analysis

Serum TNF-*α*, endotoxin, ET-1, IL-1*β*, creatine kinase-MB (CK-MB), and cardiac troponin I (cTnI) were detected using enzyme-linked immunosorbent assay (ELISA) kits (Nanjing Jiancheng Bioengineering Institute, China) according to the manufacturer's protocols. The serum levels of amylase (AMY) and lactate dehydrogenase (LDH) were measured according to the manufacturer's instructions, using an automatic biochemistry analyzer (TC6010L; Jiangxi Tecom Science Corporation, Jiangxi, China).

### 2.6. Oxidative Stress and NADPH Oxidase Activity Detection

To determine the degree of oxidative stress in the heart, lipid peroxidation (LPO), CuZn-superoxide dismutase (CuZnSOD), Mn-superoxide dismutase (MnSOD), and reduced glutathione (GSH) were measured in heart homogenates. 0.1 g of heart tissue was collected and homogenized in ice-cold normal saline (weight/volume = 1 : 9). The homogenates used for the determination of SOD activities and LPO and GSH levels were centrifuged at 3000 rpm for 10 min at 4°C, and the supernatants were collected. The SOD activities and LPO and GSH levels in the samples were measured with test kits (Nanjing Jiancheng Bioengineering Institute, China) and are presented as activity units per mg of protein (units/mg proteins).

To detect the myocardial NADPH oxidase activity, 0.5 g of heart tissue was collected and cleaned once by adding reagent buffer, then the tissue was put in cryogenic vials with liquid nitrogen and kept overnight. Next day, the tissue was ground as soon as possible into powder, then lysis buffer was added for 30 min under ice-cold conditions. After centrifugation at 10000*g* for 10 min at 4°C, the supernatants were collected. The NADPH oxidase activity in the samples were measured with test kits (Nanjing Jiancheng Bioengineering Institute, China) and are presented as activity units NADPH per min per mg of protein. Protein quantification was performed using the Bradford method. All procedures were performed in accordance with the manufacturer's instructions.

### 2.7. Detection of ROS Generation by DHE Fluorescence Staining

ROS can oxidize DHE, forming ethidium bromide to intercalate DNA. When that occurs, the compound emits red fluorescence. Intracellular ROS production in H9C2 cardiomyocytes and frozen rat heart tissues was analyzed using DHE. H9C2 cardiomyocytes and myocardium cross sections (10 *μ*m) were incubated with DHE (5 *μ*M) in PBS or high-glucose DMEM (500 : 1) in a light-protected incubator at 37°C for 30 min. The sections or cardiomyocytes were washed 3 times with PBS to remove excess DHE, and red fluorescence was assessed by a fluorescence microscope (IX81; Olympus, Tokyo, Japan) with a green light; the ROS content increased in proportion to the intensity of red fluorescence. Quantitative analysis of fluorescent images was performed with ImageJ (NIH, USA) software and expressed as arbitrary units of fluorescence.

### 2.8. Immunohistochemistry

Nox2 and Nox4 protein expression were detected in the heart tissue by immunohistochemistry as described previously [27]. The paraffin-embedded heart tissue sections were dewaxed according to standard procedures. Subsequently, the sections were dipped in a solution containing 0.01 mol/L citric acid for 5 min at 100°C, blocked with 5% bovine serum (Boster Biological Technology Co. Ltd., Wuhan, China) for 30 min, and then incubated with rabbit anti-Nox2 (1 : 1000; Abcam) and anti-Nox4 (1 : 200; Boster Biological Technology Co. Ltd.) overnight at 4°C. Sections were then incubated with biotin-labeled goat and anti-rabbit immunoglobulin G (Boster Biological Technology Co. Ltd.) for 30 min at 37°C and horseradish peroxidase-labeled streptavidin (Boster Biological Technology Co. Ltd.) for 30 min at 37°C. Finally, the specimens were stained with diaminobenzidine and nuclear counterstained with hematoxylin; then, images were captured under a Leica DM3000 microscope (Leica Microsystems CMS GmbH, Wetzlar, Germany). The brown-stained area represents cells that contain Nox4 and Nox2. The positively stained cells were observed under a light microscope and evaluated by two pathologists in a blind manner.

### 2.9. TUNEL Assay

Myocardium frozen sections (10 *μ*m) were used to detect the apoptotic myocardial cells with a TUNEL assay kit (fluorescein in situ cell death detection kit; Boster Biological Technology Co. Ltd., Wuhan, China). According to the manufacturer's instructions, all of the cells showed blue nuclear DAPI staining, but the TUNEL-positive cells displayed green nuclear staining. The stained slices were analyzed by laser-scanning confocal microscopy (Eclipse Ti2; Nikon Instruments, Tokyo, Japan).

### 2.10. Cell Viability Assay

Equal numbers of embryonic rat heart-derived H9C2 cells (Cat 6110, Cell Biology, Shanghai, China) were plated into each well of a 96-well plate. Thereafter, 5% and 10% normal or SAP rat sera were added to the culture medium, respectively, and cells were cultured for 3 h, 6 h, 12 h, and 24 h, with each group consisting of three samples. Subsequently, 10 *μ*l of the Cell Counting Kit-8 (CCK-8) solution was added to each well and the cells were incubated for 2 h. Then, the cell viability was quantified by detecting the absorbance value at 450 nm using a microplate absorbance reader (Multiskan; Thermo Fisher Scientific, Waltham, MA, USA). Each group consists of three samples.

### 2.11. Cell Culture and Flow Cytometry Analysis

A stock of H9C2 cardiomyocytes was cultured with high-glucose DMEM that was supplemented with 10% fetal bovine serum (FBS), 50 U/mL penicillin, and 50 *μ*g/mL streptomycin at 37°C in a regular plastic petri dish with an atmosphere of 95% air and 5% CO_2_. The medium was replaced every 2-3 days. Prior to experiments, cells were serum starved for 6 hours which was done in 2.5% FBS.

### 2.12. Flow Cytometry Analysis

Apoptosis was assessed with the Annexin V FITC/PI detection kit (Beijing Solarbio Science and Technology Co. Ltd., Beijing, China) according to the manufacturer's instructions. Briefly, H9C2 cardiomyocytes were seeded in six-well plates and incubated with NADPH (100 *μ*M) for each group. Apocynin (100 *μ*M), culture solution of SAP rat serum, or normal rat serum was added for 12 h. Next, cells were collected and resuspended in 100 mL binding buffer; then, 5 *μ*L of Annexin V FITC was added for 10 min and 5 *μ*L of PI was added for 5 min at room temperature in the dark, respectively. After that, 1 mL of PBS was made and cells were subjected to flow cytometry (CyFlow; Partec, Nürnberg, Germany). Experiments were repeated three times.

### 2.13. Western Blot

The proteins from the rat left-ventricular tissue or cells were extracted using a protein extraction kit (Nanjing Jiancheng Bioengineering Institute, China). The protein concentrations were determined using an enhanced BCA Protein Assay Kit (Nanjing Jiancheng Bioengineering Institute, China). Equal amounts of protein for each sample were separated by SDS-PAGE in a minigel apparatus (Mini-PROTEAN II; Bio-Rad, Hercules, CA, USA). Then, they were transferred to a 0.22 or 0.40 *μ*m PVDF membrane. Membranes were blocked with 5% milk or BSA in Tris-buffered saline-Tween 20 and were incubated overnight at 4°C with anti-Nox2 (1 : 2000), anti-Nox4 (1 : 5000), anti-Bax (1 : 2000), anti-Bcl-2 (1 : 1000), anti-caspase-3 (1 : 1000), anti-ERK1/2 (1 : 10000), anti-p-ERK1/2 (1 : 5000), anti-p38 (1 : 5000), anti-p-p38 (1 : 1000), anti-JNK (1 : 500), anti-p-JNK (1 : 1000), and anti-GADPH (1 : 5000; loading control) antibodies. After incubation with an HRP-conjugated secondary antibody, a chemiluminescence detection reagent (Millipore, Billerica, MA, USA) was added dropwise onto the membranes. Then, the membranes were examined with a BioSpectrum4 apparatus (UVP; Upland, CA, USA). The band intensities were analyzed by UVP Image Acquisition and Analysis software.

### 2.14. Statistical Analysis

All data are presented as mean ± SD and analyzed using SPSS 18 statistical software (SPSS Inc., Chicago, IL, USA). Comparisons among the different groups were determined by one-way analysis of variance, and *p* < 0.05 was recognized as statistically significant.

## 3. Results

### 3.1. NADPH Oxidase Activity in SAP-Induced Myocardial Injury

**Figure 1:**
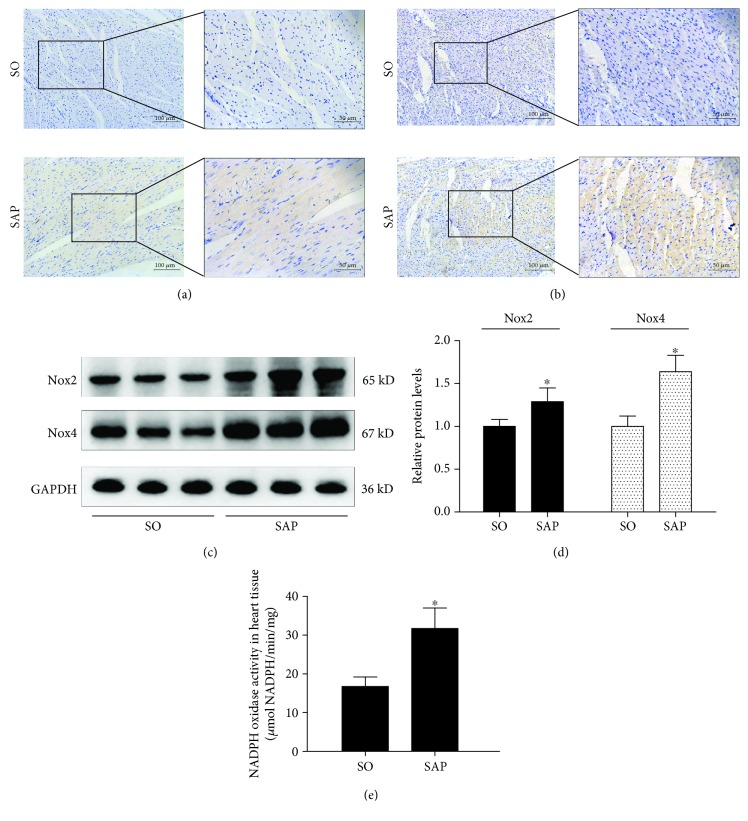
Nox activity in myocardial tissues in SAP rats. Representative immunohistochemistry images of Nox2 (a) and Nox4 (b) in myocardial tissue are shown 24 h after the induction of SAP. Immunoblot of myocardial Nox2 and Nox4 protein expressions (c). Quantitative densitometric analyses of the immunoblot data. GAPDH was used as internal control (d). Nox activity was measured by colorimetric method in heart tissue (e). Data are expressed as means ± SD, *n* = 3 rats per group. ^∗^ *p* < 0.05 compared with the SO group.

To investigate whether Nox is activated in the myocardium in SAP rats, we first determined the changes in the protein levels of Nox2 and Nox4 in myocardial tissue 24 h after SAP induction, as shown in Figure 1. Immunohistochemistry analysis showed that positive staining for Nox2 and Nox4 in the myocardium was significantly increased in the SAP group compared with the SO group, reflecting the upregulated expression of Nox2 and Nox4 in the SAP group. This result was further confirmed by western blot analysis in which there was a remarkable increase in the protein levels of Nox2 and Nox4 in the SAP group compared with those of the SO group in myocardial tissue. Corresponding with the increased protein expression of Nox, the activity of Nox was enhanced in the SAP group compared with that in the SO group in the heart (Figure 1(e)). These data demonstrated that Nox2 and Nox4 were indeed activated in the myocardium, suggesting that Nox was involved in SAP-induced myocardial injury.

### 3.2. NADPH Oxidase Hyperactivity Correlates with Exacerbated Oxidative Stress in SAP-Induced Cardiac Injury

**Figure 2:**
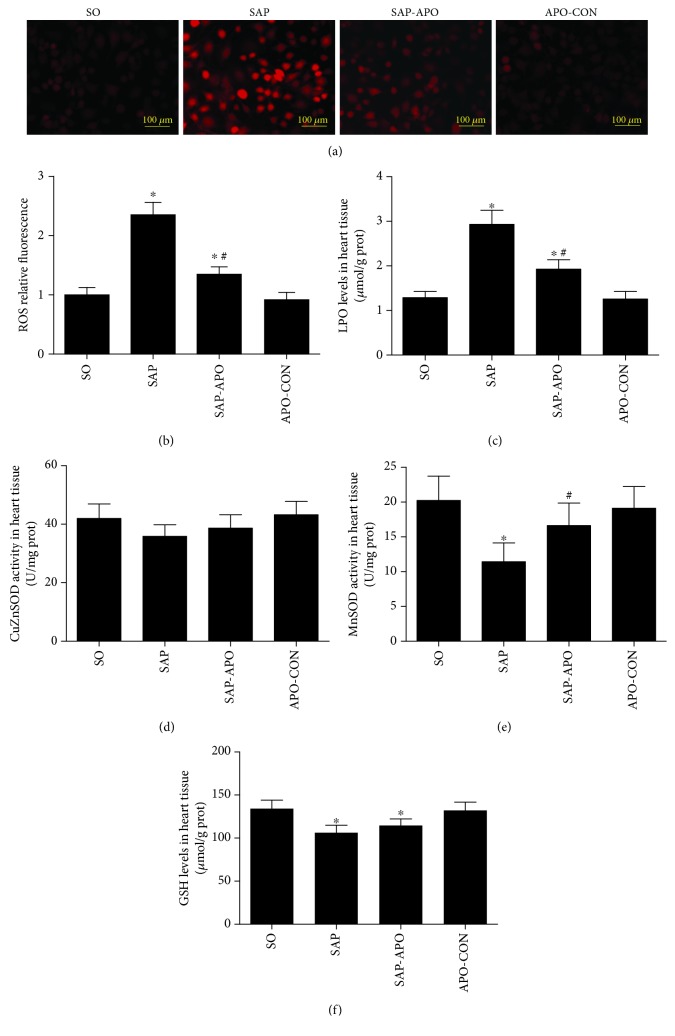
Nox hyperactivity correlated with exacerbated oxidative stress in SAP-induced cardiac injury. Effect of Nox inhibition on ROS production and oxidative indexes. DHE staining in frozen sections by fluorescent microscopy (100x magnification) (a). The fluorescence values are expressed as the ratio to the levels in the sham group (b). The levels of LPO (c) and GSH (f) and the activity of CuZnSOD (d) and MnSOD (e) in heart tissues. Data are expressed as means ± SD, *n* = 6 rats per group. LPO, lipid peroxidation; SOD, superoxide dismutase; GSH, reduced glutathione. ^∗^ *p* < 0.05 compared with the SO group. ^#^ *p* < 0.05 compared with the SAP group.

Given that Nox function mainly generates ROS, we thus determined the ROS production by Nox (Figure 2). As assessed by DHE oxidation, ROS production was increased in the myocardium in the SAP group when compared with the SO group, suggesting a correlation between increased ROS production and Nox hyperactivity. This result was further evidenced by significantly elevated LPO levels and decreased MnSOD activity and GSH levels in the SAP group compared to those in the SO group shown in Figures 2(c), 2(e), and 2(f). To confirm whether there is a direct correlation between increased ROS production and Nox hyperactivity, Nox was inhibited by treating rats with apocynin before SAP induction. Interestingly, markedly reduced LPO levels and enhanced MnSOD activity were observed in the SAP-APO group, while the GSH level only displayed an uptrend in the SAP-APO group. These data suggested that Nox hyperactivity was correlated with exacerbated oxidative stress in SAP-associated cardiac injury.

### 3.3. NADPH Oxidase Hyperactivity Correlates with Exacerbated Cardiomyocyte Apoptosis in SAP-Induced Cardiac Injury

**Figure 3:**
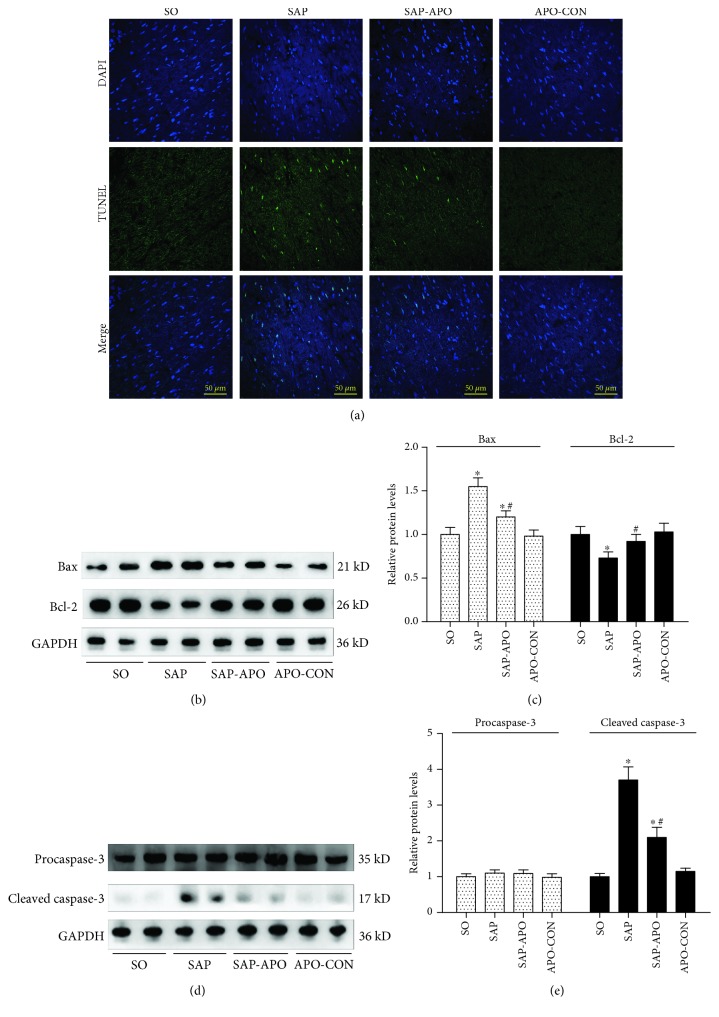
Nox hyperactivity contributes to cardiomyocyte apoptosis in SAP rats. Nox inhibition attenuates myocardial cell apoptosis and apoptosis-associated protein expression in the myocardium 24 h after SAP challenge. Representative images (400x magnification) of TUNEL assay (a). Immunoblot of Bax and Bcl-2 (b) and procaspase-3 and cleaved-caspase-3 (d) protein expressions from heart samples. Densitometry analysis of Bax and Bcl-2 (c) and procaspase-3 and cleaved-caspase-3 (e). Data are expressed as means ± SD, *n* = 3 fluorescence values per group; means ± SD, *n* = 3 immunoblot results per group. ^∗^ *p* < 0.05 compared with the SO group. ^#^ *p* < 0.05 compared with the SAP group.

As reported in the literature, exacerbated oxidative stress can cause cardiomyocyte apoptosis in various cardiac diseases. Therefore, we next evaluated the changes of cell apoptosis in the myocardium in SAP rats by TUNEL staining. As shown in Figure 3(a), the apoptotic index was significantly higher in the SAP group (26.78 ± 6.54 versus 0.00 ± 0.00, *p* < 0.05) as compared to the SO group. However, apocynin treatment induced a significant decrease in the incidence of cell apoptosis (8.35 ± 2.19 versus 26.78 ± 6.54, *p* < 0.01) compared with the SAP group. Moreover, western blotting showed that Nox inhibition downregulated the levels of proapoptotic Bax and cleaved caspase-3 and upregulated the anti-apoptotic Bcl-2 levels (Figures 3(b)–3(e)). These results suggested that Nox hyperactivity contributed to cardiomyocyte apoptosis in SAP rats.

### 3.4. NADPH Oxidase Inhibition Attenuates Cardiac Injury and Improves Cardiac Function

**Figure 4:**
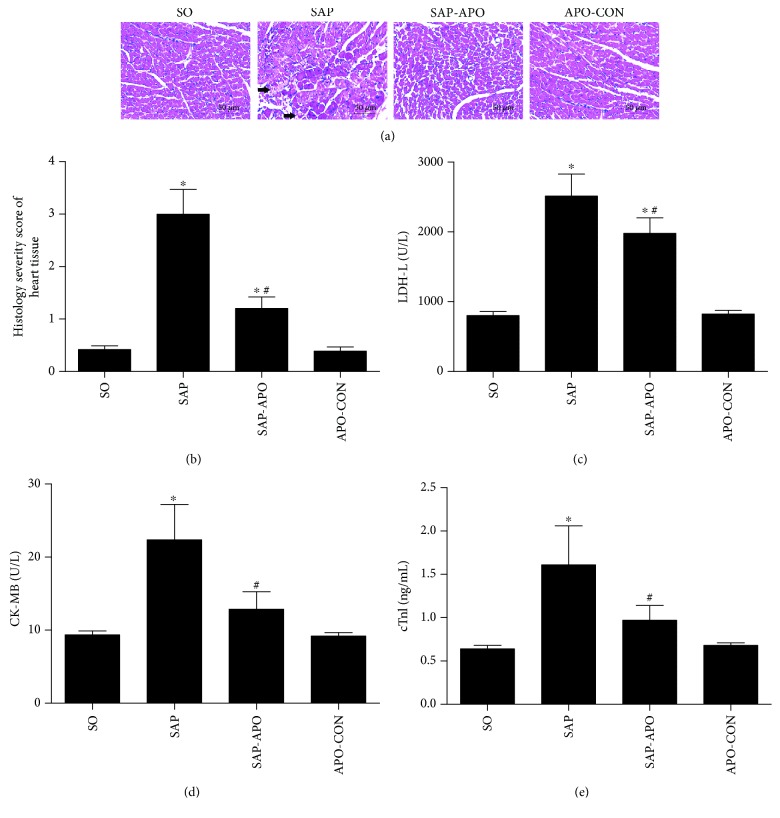
Effects of Nox inhibition on cardiac histopathology and cardiac-related enzymes 24 h after SAP induction. Representative micrographs of HE-stained sections of rat heart tissue from different groups; the arrow shows disruptive myocardial fibers. Images were taken under 200x magnification (a). Histology severity score of heart (b). Lactic dehydrogenase-L (LDH-L) (c). Creatine kinase isoenzyme MB (CK-MB) (d). Cardiac troponin I (cTnI) (e). Data are presented as mean ± SD, *n* = 6 rats per group. SO, sham operation; SAP, severe acute pancreatitis; SAP-APO, SAP+apocynin; APO-CON, apocynin control. ^∗^ *p* < 0.05 compared with the SO group. ^#^ *p* < 0.05 compared with the SAP group.

In light of the previously described results, we sought to determine the contribution of Nox on SAP-induced cardiac injury. Rats were administered with or without apocynin before SAP induction, and the changes in cardiac morphology, cardiac-related enzymes, and echocardiogram were performed. As shown in Figure 4(a), the SO group showed normal myocardial architecture. In contrast, SAP rats exhibited obvious structural and cellular changes in the myocardium, including disruptive myocardial fibers, cellular edema, and intensive infiltration, suggesting that myocardial pathological damage occurred in the SAP group. Interestingly, apocynin treatment improved histological changes in the myocardium and remarkably decreased the histopathological scores compared with the SAP group. These morphological changes were further supported by cardiac-related enzymes like LDH-L, CK-MB, and cTnI (Figures 4(c)–4(e)), which are the sensitive and specific indexes to reflect cardiac injury. There was a significant increase in cardiac-related enzymes in serum exhibited in the SAP group compared to the SO group. However, the levels of these cardiac enzymes in the SAP-APO group were significantly lower compared with those in the SAP group (*p* < 0.01), especially CK-MB and cTnI. These results indicated that Nox inhibition attenuated myocardial injury in SAP rats.

**Figure 5:**
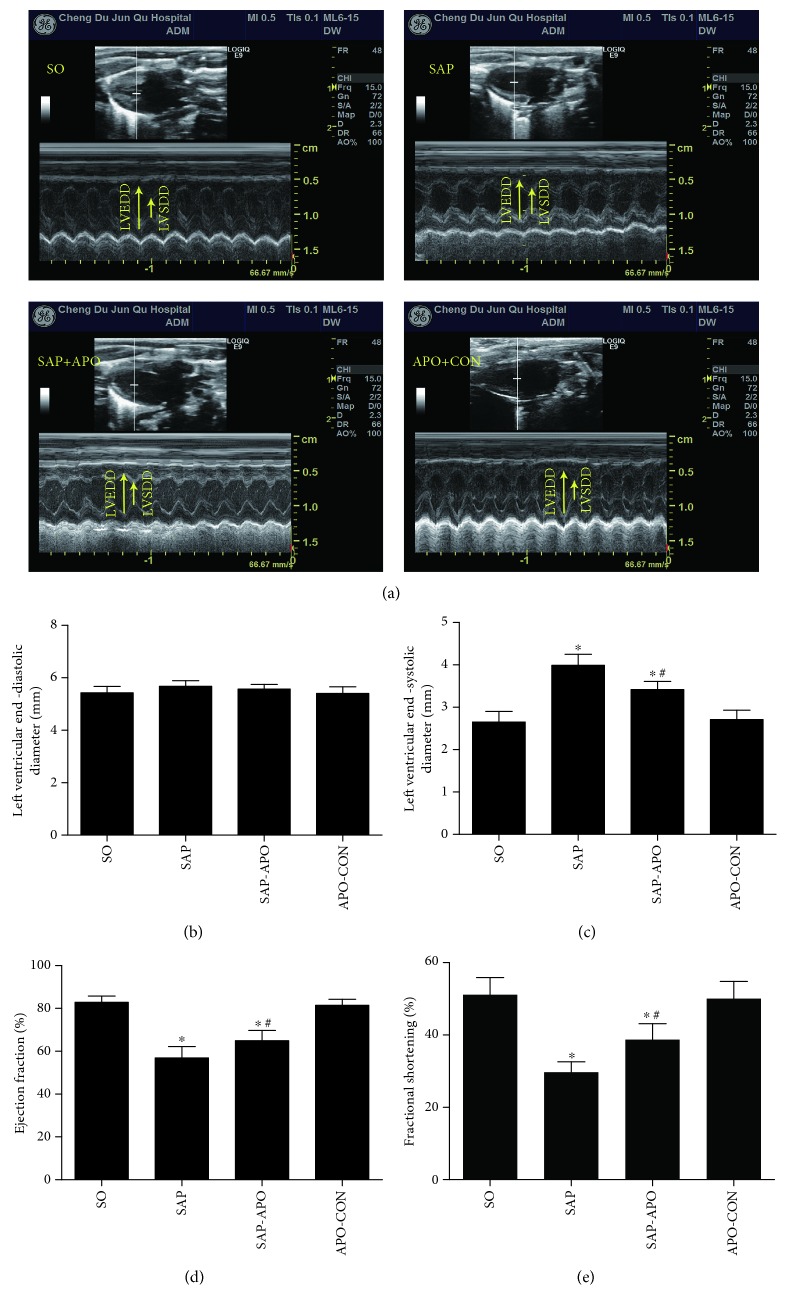
Nox inhibition attenuates echocardiographic properties after a 24 h SAP challenge. Representative M-mode images in groups (a). Left ventricular end-diastolic diameter (mm) (b). Left ventricular end-systolic diameter (mm) (c). Ejection fraction (%) (d). Fractional shortening (%) (e). Data are presented as mean ± SD, *n* = 6 rats per group. ^∗^ *p* < 0.05 compared with the SO group. ^#^ *p* < 0.05 compared with the SAP group.

To further evaluate the effect of Nox inhibition on SAP-induced myocardial injury, cardiac function and hemodynamic changes were measured 24 h after SAP challenge by using M-mode echocardiography and a polygraph system. As seen in Figure 5 and Figure S1, Nox inhibition significantly decreased LVESD (left ventricular end-systolic dimension) and increased the FS, EF (ejection fraction), and systolic pressure compared with the SAP group. Despite that there was no statistical significance in heart rate and systolic pressure between the SAP and SAP-APO groups, an uptrend existed in SAP rats pretreated with apocynin. These findings indicated that Nox inhibition attenuated cardiac dysfunction and hemodynamic changes in SAP rats.

**Figure 6:**
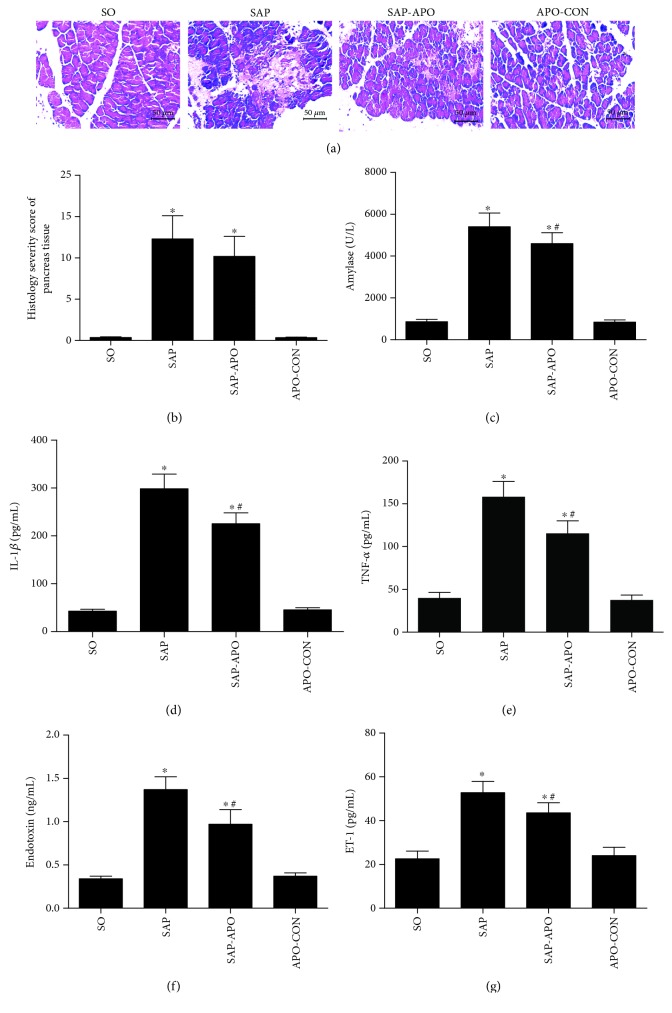
Effects of Nox inhibition on pancreatic histopathology and proinflammatory cytokines after 24 h SAP induction. Representative micrographs of HE-stained sections of rat pancreatic tissue from different groups; images were taken under 200x magnification (a). Histology severity score of pancreas (b). Amylase (U/L) (c). Interleukine-1 beta (IL-1*β*) (d). Tumor necrosis factor alpha (TNF-*α*) (e). Endotoxin (ng/mL) (f). Endothelin-1 (ET-1) (g). Data are presented as mean ± SD, *n* = 6 rats per group. SO, sham operation; SAP, severe acute pancreatitis; SAP-APO, SAP+apocynin; APO-CON, apocynin control. ^∗^ *p* < 0.05 compared with the SO group. ^#^ *p* < 0.05 compared with the SAP group.

Additionally, considering that the key feature of acute pancreatitis is damage to the pancreas, we evaluated the role of Nox inhibition on pancreatic injury in SAP. As shown in Figure 6, pancreatic injury in SAP rats manifested as a large area of tissue necrosis, elevated amylase activity, and proinflammatory cytokines as reflected by serum levels of IL-1*β*, TNF-*α*, endotoxin, and ET-1. Apocynin treatment exhibited a declining trend in histopathological scores and markedly decreased levels of amylase activity, IL-1*β*, TNF-*α*, endotoxin, and ET-1 compared with the SAP group. However, the concentrations of these indexes in the SAP-APO group were still much higher than those in the SO group (*p* < 0.05). These observations indicated that Nox inhibition had limited protective effect on pancreatic injury in SAP rats.

### 3.5. NADPH Oxidase Inhibition Exerts a Beneficial Effect via Downregulating MAPK Pathway In Vivo

**Figure 7:**
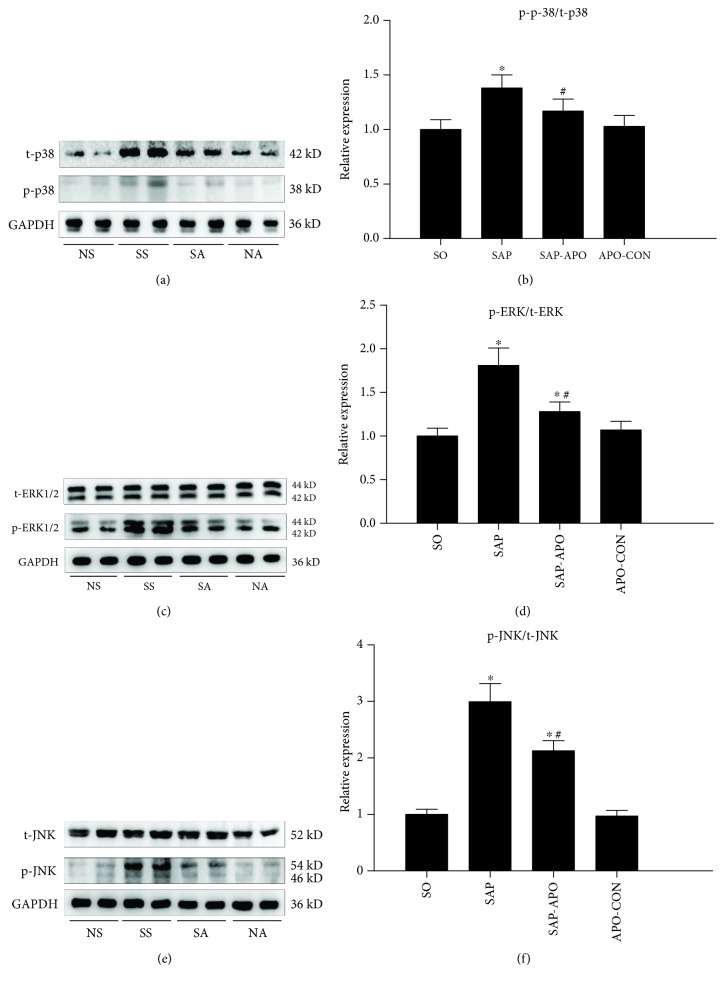
The beneficial effect of Nox inhibition via downregulating the MAPK pathway in vivo. Representative immunoblots of heart tissue p38 (a), ERK (c), and JNK (e) protein expressions and their phosphorylation levels. The blots were analyzed by densitometry, and the results are expressed in the histograms (b, d, and f, respectively). Data are expressed as means ± SD, *n* = 3 immunoblot results per group. ^∗^ *p* < 0.05 compared with the NS group. ^#^ *p* < 0.05 compared with the SS group.

Since Nox inhibition exerted a protective role on oxidant stress damage and cell apoptosis in SAP-induced cardiac injury, we further explore the underlying molecular mechanism of this protection. The redox-sensitive MAPK signaling pathways are usually reported to regulate the inflammation and apoptosis of cardiomyocytes, and thus their signaling molecules including JNK, ERK, and p38 were examined by western blot analysis. As presented in Figure 7, the phosphorylation levels of ERK, p38, and JNK were increased significantly in the SAP group compared with that in the SO group, indicating that MAPK signaling pathways are involved in SAP-induced cardiac injury. Interestingly, with the pretreatment of apocynin, the increased phosphorylation levels of JNK, ERK, and p38 were found to be significantly downregulated. These data indicated that SAP-induced increases in JNK, ERK, and p38 in heart tissue were significantly prevented by pretreatment with apocynin.

### 3.6. NADPH Oxidase Inhibition Reduces Oxidative Stress and Suppresses Cell Apoptosis in an In Vitro Simulation Experiment

**Figure 8:**
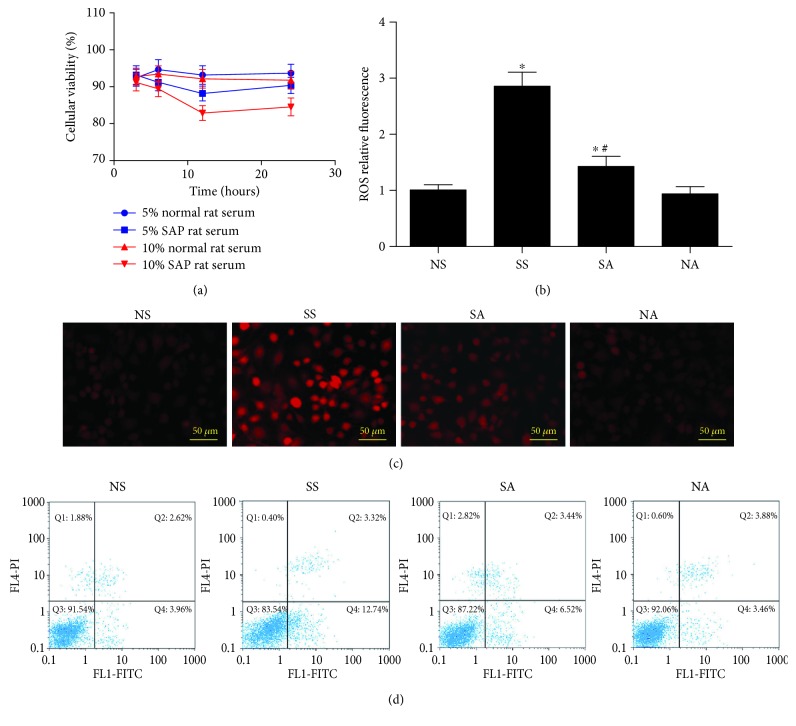
The effects of Nox inhibition on oxidant stress damage and cell apoptosis in mimic SAP-induced cardiac injury *in vitro*. The CCK-8 assay shows that 10% SAP rat serum was the appropriate concentration and 12 h was the suitable time (a). Nox inhibition attenuates ROS production and apoptosis of H9C2 cardiomyocyte stimulated by SAP serum *in vitro*. Intracellular ROS level was detected by using DHE staining. The fluorescence values are expressed as the ratio to the levels in the NS group cells (b and c). Flow cytometry analysis of apoptosis in H9C2 cardiomyocytes by using Annexin V FITC/PI double staining (d). NS, normal rat serum group; SS, SAP rat serum group; SA, SAP rat serum and apocynin group; NA, normal rat serum and apocynin group. ^∗^ *p* < 0.05 compared with the NS group. ^#^ *p* < 0.05 compared with the SS group.

To further confirm the effects of Nox inhibition on SAP-induced cardiac injury, H9C2 cardiomyocytes were cultured *in vitro* with the addition of SAP serum to simulate the cardiomyocytes in SAP rats; these were then compared to those in normal rat serum. Firstly, to determine the optimal concentration and action time, we first evaluated the viability of SAP serum with different concentrations (5% and 10%) on cardiomyocytes within 24 h by CCK-8 assay. As shown in Figure 8(a), the survival rate of H9C2 cells displayed a marked decrease at 12 h when the concentration of SAP serum was 10%. Thus, we chose the 10% concentration of SAP serum and 12 h of action time for cardiomyocyte culture and for further investigation.

Next, we investigated the changes of ROS production and cell apoptosis when cardiomyocytes were exposed to SAP serum. As seen in Figure 8(c), strong red fluorescence was found in cardiomyocytes after exposure to SAP serum, suggesting the increased ROS production in cardiomyocytes. In contrast, apocynin pretreatment significantly declined ROS levels in cardiomyocytes after exposure to SAP serum. Furthermore, flow cytometry analysis showed that significant apoptosis was noted in H9C2 cardiomyocytes after exposure to SAP serum (17.06 ± 2.83) when compared with other groups (6.69 ± 0.36 in the NS group, 11.28 ± 1.64 in the SA group, and 6.26 ± 0.31 in the NA group), which was in agreement with the results *in vivo*. However, the phenomenon was suppressed by apocynin pretreatment (Figure 8(d)). Overall, these results demonstrated that Nox inhibition reduced oxidant stress damage and cell apoptosis in mimic SAP-associated cardiac injury *in vitro*.

### 3.7. NADPH Oxidase Inhibition Reduces the Activation of MAPK Pathway in an In Vitro Simulation Experiment

**Figure 9:**
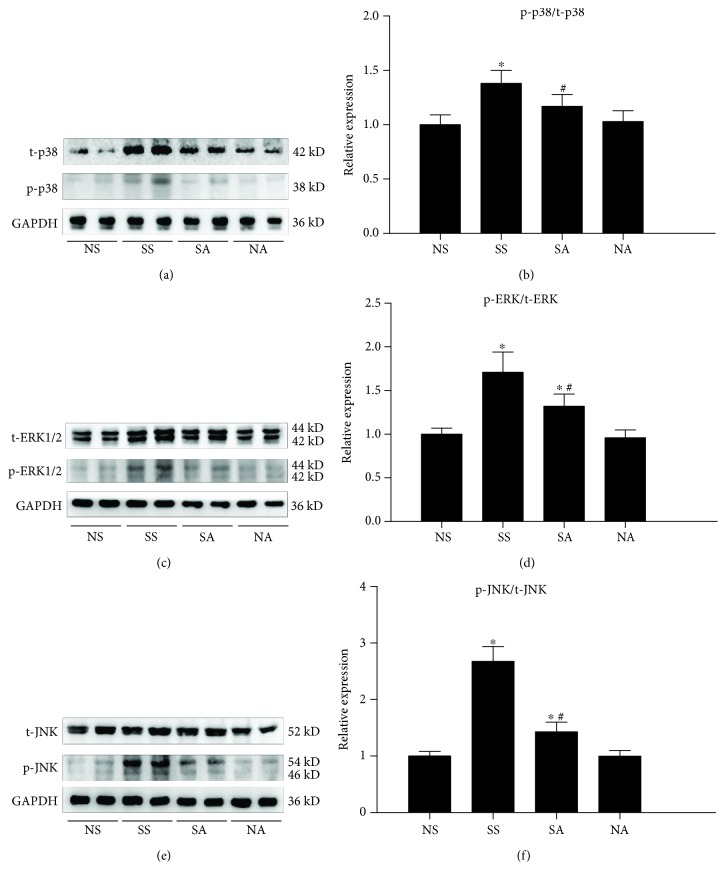
The beneficial effect of Nox inhibition via downregulating the MAPK pathway. Representative immunoblots of H9C2 cardiomyocytic p38 (a), ERK (c), and JNK (e) protein expressions and their phosphorylation levels. The blots were analyzed by densitometry, and the results are expressed in the histograms (b, d, and f, respectively). Data are expressed as means ± SD, *n* = 3 immunoblot results per group. ^∗^ *p* < 0.05 compared with the NS group. ^#^ *p* < 0.05 compared with the SS group.

To further confirm the results shown in Figure 4, we measured the expression of JNK, ERK, and p38 in an *in vitro* simulation experiment by western blot analysis. As presented in Figure 9, the phosphorylation of ERK, p38, and JNK were increased significantly in the SS group after SAP serum stimulation compared with the NS group. With the pretreatment of apocynin, the levels of phosphorylated JNK, ERK, and p38 were downregulated significantly. These results are consistent with the data of the *in vivo* experiment, verifying that the MAPK pathway was effectively blocked by apocynin in cardiomyocytes challenged by SAP serum.

**Figure 10:**
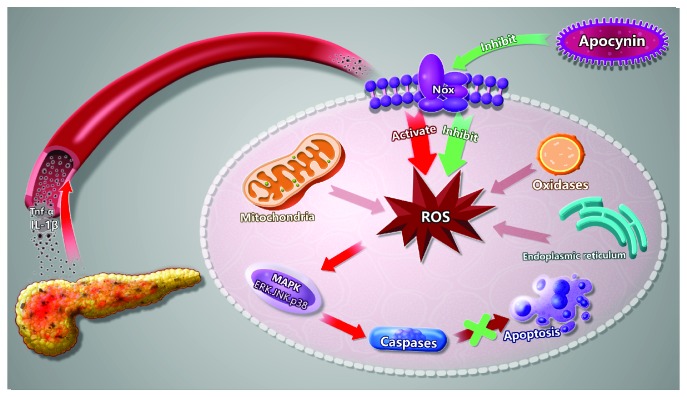
Possible mechanisms responsible for the effect of Nox on SAP-associated cardiac injury. During pancreatitis, a large number of proinflammatory cytokines and toxic substances are released into bloodstream, which can exert stimulation to Nox in the myocardial cell. The hyperactivity of Nox results in the overproduction of ROS and activates the redox-sensitive MAPK signaling pathways. These events upregulate the expression of caspase-associated proteins, which ultimately induces cell apoptosis. Pretreatment with apocynin to inhibit the activity of Nox can lead to the decrease in ROS production under SAP conditions, thus alleviating the activation of downstream signaling pathways and thereby affording beneficial effects.

Based on these findings, a schematic model was proposed to elucidate the possible mechanism responsible for the effect of Nox on SAP-induced cardiac injury (Figure 10). Once SAP occurs, Nox acts as the main source of ROS production in the myocardium, inducing oxidative stress and promoting cell apoptosis via activating the MAPK pathway, which ultimately results in cardiac injury in SAP. However, when Nox was inhibited by apocynin treatment, ROS production markedly decreases, which can effectively inhibit cell apoptosis via downregulating the MAPK pathway and thus protect against SAP-induced cardiac injury.

## 4. Discussion

To the best of our knowledge, this study demonstrates for the first time the contribution of NADPH oxidase to SAP-associated myocardial injury in rats. Our key finding was that the hyperactivity of NADPH oxidase underlies myocardial injury in SAP rats by promoting ROS generation with increased oxidative stress and cardiomyocyte apoptosis via activating the MAPK pathway, ultimately resulting in cardiac injury in SAP. These findings provide new insight into the pathogenic mechanism of SAP-associated myocardial injury, which may offer a potential therapeutic and preventive target for this complication.

SAP is a fatal clinical condition and is usually associated with multiple organ injuries [28]. Among the multiple organ injuries associated with SAP, cardiac injury is an important part of the pertinent manifestations of cardiovascular system complications, and cardiac decompensation even causes death [4, 29]. Alterations in cardiac function and myocardial lesions can be observed during SAP [30, 31]. In the present study, we established a well-characterized SAP model by injecting sodium taurocholate as previously reported with a little modification [22], and we assessed the extent of SAP-evoked cardiac injury. As expected, our results showed that SAP rats exhibited significant cardiac impairment, as evidenced by histological and echocardiographic abnormality, elevated cardiac enzymes, increased lipid peroxidation production, and apoptotic myocardial cells. Our data from the current study showed that the SAP model with cardiac injury was well established, which was in line with other reports [32, 33]. Meanwhile, plasma TNF-*α*, IL-1*β*, ET-1, and endotoxin levels were increased in the SAP group when compared with sham rats, suggesting that myocardial injury occurs along with systemic inflammation.

The increase in ROS production in cardiac cells and the accompanying oxidative stress are major initiators of cardiovascular injury [34, 35]. Nox is reported to be a major source of ROS, and Nox-mediated ROS generation in the heart increases in response to various stimuli and plays an important role in different cardiovascular diseases [36]. Since free radicals are generated excessively during SAP [37, 38], we speculated that Nox may be involved in the development of SAP-associated cardiac injury. In line with our main hypothesis, SAP rats displayed Nox hyperactivity paralleled by increased Nox2 and Nox4 protein levels when compared with sham rats during systematic inflammation stress, especially the protein expression of Nox4. SAP-induced Nox hyperactivation in the heart may be due to increased circulating proinflammatory cytokines like TNF-*α* and IL-1*β*, which have been demonstrated to be increased in SAP rats [39] and were also observed in our study. Along with SAP-induced Nox hyperactivation, we observed the increased ROS and LPO levels and decreased MnSOD activity and GSH levels in SAP rats. Notably, such redox status imbalance was inhibited by treating rats with apocynin before SAP induction, suggesting that there is a direct correlation between increased oxidative stress and Nox hyperactivity in SAP-associated cardiac injury models.

Higher levels of oxidative stress are known to promote apoptosis, which is thought to play an important role in the development of heart failure [40]. A previous study has demonstrated that parthenolide-induced Nox activation and oxidative stress are involved in decreased cell viability in cultured neonatal ventricular myocytes [41]. Another study has shown that Nox activation and apoptosis occur in H9C2 cells after exposure to angiotensin II [42]. In the present study, we detected that cell apoptosis in the myocardium increased significantly in the SAP model by TUNEL staining. The expression of apoptosis-associated protein Bax, Bcl-2, and cleaved caspase-3 also changed correspondingly. In particular, cleaved caspase-3, the final effect factor of the caspase cascade that induces apoptosis [43], was remarkably elevated. When rats were treated with apocynin before SAP induction, significant decreases in the rate of apoptosis and proapoptotic protein levels were notable in myocardial tissues. In an *in vitro* experiment, we further confirmed the protective effect of Nox inhibition on cardiomyocyte apoptosis by culturing H9C2 cardiomyocytes when exposed to SAP serum. These data demonstrated that Nox-derived ROS contribute to cardiomyocyte apoptosis submitted to SAP, suggesting that Nox has an important contribution to cardiomyocyte apoptosis in SAP models.

In the present study, we found that Nox inhibition markedly improved histological changes in the heart tissues and reversed the alterations in serum cardiac-related enzymes and cardiac function, suggesting an important contribution of Nox-mediated oxidative stress in the rat heart injury of our experimental model. Once the heart is injured, CK-MB and troponin-I (specific cardiac injury indexes) will be released into the circulation [44]. The serum levels of LDH, CK-MB, and troponin-I in SAP rats significantly declined following Nox inhibition, indicating the cardioprotective effect of Nox inhibition. In line with these findings, morphological changes indicating cardiac injury in SAP rats, including disruptive myocardial fibers, cellular edema, and intensive infiltration, were markedly alleviated after apocynin pretreatment. This protective role in cardiac injury was further confirmed by improved cardiac function and hemodynamic changes, in which Nox inhibition markedly increased FS, EF, and systolic pressure and decreased LVESD level. A previous report has demonstrated that Nox contributes to the development of cardiac contractile dysfunction in response to pressure overload, even though it is not essential for the development of LV hypertrophy per se [45]. Similar with SAP, in animal models of sepsis Nox was also found to be involved in cardiac injury [13]. These reports are in accordance with our present results manifesting that Nox inhibition attenuates myocardial injury and improves cardiac function. However, the protection for pancreatic injury was limited, in which the levels of proinflammatory cytokines were still much higher than those in the SO group. The possible explanation is that SAP is also a kind of severe disease associated with distant organ lesions so that apocynin alone is insufficient for preventing pancreatic injury. Even so, these results likewise indicated that antioxidant treatment exerts a beneficial effect against SAP-associated myocardial injury.

The MAPK family is involved in a diverse number of regulatory processes including response to growth signals, apoptosis, and sensitivity to stress [46]. JNK, ERK1/2, and p38 MAPK are the major subfamilies of MAPK signaling pathways, and it has been confirmed that the MAPK signaling pathway participates in cardiac diseases. Nox activation mediated ROS production in cardiac failure in parallel with the activation of MAPK, and it is reported to be implicated in myocyte apoptosis [20, 21]. In our study, we observed a significant alteration of the JNK, ERK1/2, and p38 MAPK phosphorylation levels when the heart is exposed to SAP stress, suggesting that MAPK pathways are activated in myocardial cells in the inflammatory state. To further confirm the findings, we conducted a simulation experiment with H9C2 cardiomyocytes *in vitro*. When cardiomyocytes were exposed to SAP serum, the expression of JNK, ERK, and p38 obviously increased. This result is consistent with previous reports that increased ERK1/2 and p38 phosphorylation levels were observed in neonatal cardiomyocytes challenged by LPS *in vitro* [19]. Interestingly, once Nox was inhibited by apocynin, the phosphorylation levels of JNK, ERK1/2, and p38 were significantly decreased in the *in vivo* and *in vitro* study when compared with the SAP challenged group. Apocynin suppresses the phosphorylation of p38, JNK, and ERK1/2 through its inhibitory effect on Nox activity. These results imply that MAPK signaling pathways play important roles in Nox-mediated cardiac injury in SAP.

Our study has several limitations. Firstly, we mainly focus on the role of Nox in SAP-associated cardiac injury, but whether Nox is also involved in other distant organ injuries needs to be studied further. Secondly, as to which kind of inflammatory factor activates Nox and what kind of activation process occurs during SAP are still unknown; a future study should be carried out. Thirdly, apocynin was employed to exclude ROS production by Nox; however, it must be noted that apocynin is a nonspecific inhibitor of Nox. In future experiments, we should utilize a specific inhibitor of Nox or a special isoform knockout model to clarify the precise mechanisms underlying the antioxidative protection effect for SAP-induced myocardial injury.

## 5. Conclusions

In summary, our study found that NADPH oxidase hyperactivation acts as the main source of ROS production in the myocardium that enhances oxidative stress and promotes cardiomyocyte apoptosis via activating MAPK signaling pathways, ultimately resulting in cardiac injury in SAP. Nox inhibition with apocynin suppresses the activation of MAPK signaling pathways and reduces cardiac injury. Our finding suggests that the Nox inhibitor exerts a cardioprotective effect in SAP, which may provide new therapeutic potential to prevent SAP-associated cardiac injury.

## Data Availability

The data used to support the findings of this study are available from the corresponding author upon request.

## Abbreviations

NADPH: nicotinamide adenine dinucleotide phosphate
SAP: severe acute pancreatitis
LPO: lipid peroxidation
SOD: superoxide dismutase
GSH: reduced glutathione
ROS: reactive oxygen species
MAPKs: mitogen-activated protein kinases
ERK: extracellular regulated protein kinase
JNK: c-Jun amino-terminal kinase.

## Supplementary Materials

Supplementary Materials The effect of NADPH oxidase inhibition on heart rate and blood pressure. To evaluate the effect of Nox inhibition on hemodynamic changes, the heart rate and blood pressure were measured 24 h after SAP induction. As shown in Figure S1, heart rate, systolic pressure, and diastolic pressure declined significantly in the SAP group as compared with the SO group. With the pretreatment of apocynin, the systolic pressure was obviously improved after 24 h of SAP induction. Despite that there was no statistical significance in the heart rate and systolic pressure between the SAP and SAP-APO groups, an uptrend existed in the SAP rats pretreated with apocynin. These results indicate that Nox inhibition attenuates hemodynamic changes in SAP rats. Figure S1: effect of Nox inhibition on heart rate and blood pressure after 24 h of SAP induction. Heart rate (A). Systolic pressure (mmHg) (B). Diastolic pressure (mmHg) (C). Data are presented as mean ± SD, *n* = 6 rats per group. ^∗^ *p* < 0.05 compared with the SO group. ^#^ *p* < 0.05 compared with the SAP group.
