# Supplementary material for: NADPH Oxidase Hyperactivity Contributes to Cardiac Dysfunction and Apoptosis in Rats with Severe Experimental Pancreatitis through ROS-Mediated MAPK Signaling Pathway
Source: Oxid Med Cell Longev. 2019 May 9;2019:4578175. doi: 10.1155/2019/4578175 (PMC6532283; doi:10.1155/2019/4578175)
Supplement: Supplementary Materials — The effect of NADPH oxidase inhibition on heart rate and blood pressure. To evaluate the effect of Nox inhibition on hemodynamic changes, the heart rate and blood pressure were measured 24 h after SAP induction. As shown in Figure S1, heart rate, systolic pressure, and diastolic pressure declined significantly in the SAP group as compared with the SO group. With the pretreatment of apocynin, the systolic pressure was obviously improved after 24 h of SAP induction. Despite that there was no statistical significance in the heart rate and systolic pressure between the SAP and SAP-APO groups, an uptrend existed in the SAP rats pretreated with apocynin. These results indicate that Nox inhibition attenuates hemodynamic changes in SAP rats. Figure S1: effect of Nox inhibition on heart rate and blood pressure after 24 h of SAP induction. Heart rate (A). Systolic pressure (mmHg) (B). Diastolic pressure (mmHg) (C). Data are presented as mean ± SD, n = 6 rats per group. ∗ p < 0.05 compared with the SO group. # p < 0.05 compared with the SAP group. [file 4578175.f1.docx]

The effect of NADPH oxidase inhibition on heart rate and blood pressure.
To evaluate the effect of Nox inhibition on hemodynamic changes, heart rate and blood pressure were measured 24 h after SAP induction
